# Comparison of tracheal and choanal cleft swabs and poultry dust samples for detection of Newcastle disease virus and infectious bronchitis virus genome in vaccinated meat chicken flocks

**DOI:** 10.1371/journal.pone.0247729

**Published:** 2021-04-16

**Authors:** Awol M. Assen, Stephen W. Walkden-Brown, Mark Stillman, Sheridan Alfirevich, Priscilla F. Gerber

**Affiliations:** 1 Animal Science, School of Environmental and Rural Science, University of New England, Armidale, New South Wales, Australia; 2 School of Veterinary Medicine, Wollo University, Dessie, Ethiopia; 3 Baiada Poultry Pty Limited, New South Wales, Australia; University of Nicolaus Copernicus in Torun, POLAND

## Abstract

This study assessed different methods (tracheal and choanal cleft swabs from individual birds, and poultry dust as a population level measure) to evaluate the shedding kinetics of infectious bronchitis virus (IBV) and Newcastle disease virus (NDV) genome in meat chicken flocks after spray vaccination at hatchery. Dust samples and tracheal and choanal cleft swabs were collected from four meat chicken flocks at 10, 14, 21 and 31 days post vaccination (dpv) and tested for IBV and NDV genome copies (GC) by reverse transcriptase (RT)-PCR. IBV and NDV GC were detected in all sample types throughout the study period. Detection rates for choanal cleft and tracheal swabs were comparable, with moderate and fair agreement between sample types for IBV (McNemar’s = 0.27, kappa = 0.44) and NDV (McNemar’s = 0.09; kappa = 0.31) GC respectively. There was no significant association for IBV GC in swabs and dust samples (R^2^ = 0.15, P = 0.13) but NDV detection rates and viral load in swabs were strongly associated with NDV GC in dust samples (R^2^ = 0.86 and R^2^ = 0.90, P<0.001). There was no difference in IBV and NDV GC in dust samples collected from different locations within a poultry house. In conclusion, dust samples collected from any location within poultry house show promise for monitoring IBV and NDV GC in meat chickens at a population level and choanal cleft swabs can be used for detection of IBV and NDV GC instead of tracheal swabs in individual birds.

## 1. Introduction

Infectious bronchitis (IB) and Newcastle disease (ND) are highly transmissible diseases that cause substantial economic losses in the poultry industry [1, 2]. Infectious bronchitis is caused by infectious bronchitis virus (IBV), a gammacoronavirus with a single-stranded positive sense RNA genome [3]. Newcastle disease is caused by virulent strains of Newcastle disease virus (NDV), a paramyxovirus with a single-stranded negative sense RNA genome [4]. Infectious bronchitis virus can be acquired by inhalation or direct contact with infected birds or contaminated litter or equipment. Regardless of tissue tropism of strains, IBV initially infectes the respiratory tract and the virus mainly replicates in the upper respiratory tract [5] causing conjunctivitis, tracheitis, and ciliostasis [6]. IBV is excreted in respiratory aerosols and excreta [5]. Birds can get infected with NDV by inhaling or ingesting contaminated dust, aerosols or feces [7, 8]. ND affects the respiratory, gastrointestinal, nervous and reproductive systems [9] and similarly to IBV, NDV is excreted in oropharyngeal secretions and excreta [10]. Newcastle disease virus is relatively stable outside of the host and infectious NDV has been shown to survive in poultry houses for up to 16 days post depopulation of infected birds [11].

Vaccine and field strains of IBV and NDV have been detected in tracheal, oropharyngeal or choanal cleft swabs for variable lengths of time post infection or vaccination. Different strains of IBV were detected in tracheal tissue [12], and tracheal [13–17], choanal cleft [13, 18, 19] and oropharyngeal swabs [20]. Similarly, NDV RNA has been detected from oral [21], oropharyngeal [22, 23] and tracheal [24] swabs. For detection of both viruses, choanal cleft and tracheal swabs could be used as diagnostic samples. Choanal cleft swabs are less invasive and easier to collect compared to tracheal swabs; however, to the best of the authors’ knowledge, a comparison on the sensitivity of detection of IBV and NDV nucleic acids for both sample types has not yet been performed.

Regardless of the sample type chosen, disease incursion or vaccine uptake monitoring in poultry flocks based on individual bird sampling requires collection of a large number of samples to achieve representativeness of the population, a process that is labour intensive and not economical in large meat chicken flocks. A method based on population level sampling for monitoring vaccine uptake or wild-type virus incursion of Marek’s disease virus using molecular testing of poultry dust has been used by the poultry industry in various countries [25–27]. More recently, this method has been used to monitor infectious laryngotracheitis virus vaccine uptake in chicken farms [28–30]. PCR-based dust testing has advantages of ease of collection by farm staff, increased bird welfare as it is a non-invasive sample, and a reduced number of samples are necessary to represent a flock compared to individual sampling. In addition, the viral genomic material is highly stable in dust with IBV genome stable for at least 4 months in dust stored up to 37°C [31], although it appears that viruses are rapidly inactivated in this sample type [32]. Dust based molecular approaches of flock screening and monitoring could be extended for other poultry viruses such as IBV and NDV.

This study aimed to provide a proof of principle on the use of a poultry dust, a population level sample, for evaluating the shedding kinetics of IBV and NDV genome in a vaccinated flock compared to tracheal and choanal cleft swabs from individual birds. The specific objectives were to 1. Determine the detection rates and load of IBV and NDV genome in tracheal and choanal cleft swabs and dust samples collected from commercial meat chicken flocks following live vaccination at hatchery; 2. Assess the agreement between tracheal and choanal cleft swabs in detecting IBV and NDV genomes; 3. Investigate the association of the detection rates of IBV and NDV genome in individual swab samples and viral load in dust samples; 4. To determine the effect of location of dust collection within poultry house on dust deposition rate and IBV and NDV detection rates and load in dust.

## 2. Materials and methods

### 2.1 Ethics statement

The experimental protocol used in this study was approved by the University of New England (UNE, Armidale, Australia) Animal Ethics Committee (AEC19-011). Swab samples were quickly collected by experienced veterinarians to ensure bird welfare. Samples used in this study were collected as part of a study on infectious laryngotracheitis virus vaccination [30].

### 2.2 Experimental design

The study was conducted in four meat chicken (Ross) flocks of a single commercial farm located in New South Wales, Australia. Birds were kept in tunnel ventilated houses having rice hulls as bedding material. Each one of the four poultry flocks had approximately 49,000 birds [30]. Live IBV (VicS strain) and NDV (V4 strain) vaccines were mixed together and administered in spray cabinet on the day of hatch at hatchery.

A total of 64 (n = 16/flock) dust samples were collected using dust collection settle plates as previously described [28]. Four dust collection plates with a surface area of 520 cm^2^ were placed at a height of approximately 1.5 m on the day of chicken placement in each house. Plates were numbered from one to four, from the farthest to the nearest to the exhaust fans. Dust samples were collected 10 days after chicken placement and then on weekly basis for three consecutive weeks, corresponding to 10, 17, 24 and 31 days post vaccination (dpv). Total amount of dust collected at each collection time was weighed and expressed as milligram of dust per 100 cm^2^ area per day. Paired tracheal and choanal cleft swabs were collected on the same days of dust collection from 20 arbitrarily selected chickens to determine the point prevalence of IBV and NDV, except on 10 dpv when only tracheal swabs were collected from five to six birds per flock. On sampling days 17, 24 and 31 dpv, choanal cleft swabs were collected from an additional 20 chickens. A total of 261 tracheal and 480 choanal cleft swabs were collected. Dust and swab samples were transported in dry ice to the laboratory and stored at -20°C until processing.

### 2.3 Nucleic acid extraction and IBV and NDV genomic detection

Dust RNA was extracted from approximately 5 mg of sample using ISOLATE II RNA Mini kit (Bioline, Australia) according to the manufacturer’s recommendations. Dust samples weighing less than 5 mg (2/64) were not processed. Swabs were cut using clean scissors into a 1.5 ml microtube containing 800 μl of sterile buffered phosphate saline. After vortexing for 10 seconds, 200 μl of swab wash was taken and RNA was extracted using GeneJET Viral DNA and RNA Purification Kit (Thermo Fisher Scientific, Australia) following manufacturer’s recommendations providing a final eluted volume of 60 μl. Extracted RNA were stored at -20°C until needed for reverse transcriptase (RT)-PCR analysis.

Extracts were tested for IBV and NDV RNA by a one-step duplex real-time RT-PCR targeting the matrix (M) gene of NDV [33] and the 5′ untranslated region (UTR) gene of IBV [34]. Standards used for quantification of IBV and NDV genome copies (GC) were constructed using target DNA templates transcribed to RNA using TranscriptAid T7 High Yield Transcription Kit (Thermo Fisher Scientific, Australia) following the manufacturer’s protocol and used to create standard curves. Viral GC were expressed as log_10_ GC per milligram of dust for dust samples and log_10_ GC per swab for swabs. The concentration per swab was calculated by multiplying the viral load per reaction by 80, i.e. ×20 (corresponding to 3μl of 60μl elution volume was used as template for PCR) then × 4 (corresponding to 200 μl of 800 μl total volume of PBS from swabs used for nucleic acid extraction).

### 2.4 Data analysis

Statistical analyses were performed using JMP software version 14 (SAS Institute, Cary, NC, USA). Viral GC values were transformed to log_10_ (log_10_ GC +1) to better meet the assumptions of the parametric analysis. Log_10_ GC of IBV and NDV per mg of dust were analysed using a restricted maximum likelihood model fitting location of dust collection (plate number) nested within flock as a random effect and dpv, location, flock and their interactions as fixed effects. Log_10_ GC of IBV and NDV per tracheal and choanal cleft swabs were analysed using a general linear model fitting dpv, flock and their interactions as fixed effects. Dust deposition rate was analysed by fitting chicken age, flock, plate location and their interaction as fixed effects in a general linear model. Discrete data such as IBV and NDV positive or negative RT-PCR results for choanal cleft and tracheal swabs at different dpv were subjected to contingency table analysis. Level of agreement in log_10_ IBV and NDV GC between paired tracheal and choanal cleft swabs was determined by intraclass correlation coefficient (ICC). ICC values < 0.5, 0.5–0.75, 0.75–0.9 and > 0.9 were considered as indicative of poor, moderate, good and excellent agreement respectively [35]. The agreement between paired tracheal and choanal cleft swabs in detecting IBV and NDV positive birds was tested by McNemar’s test. Kappa value was used to determine the strength of agreement. Values ≤ 0 were considered to have no agreement, 0.01–0.20 as slight, 0.21–0.40 as fair, 0.41–0.60 as moderate, 0.61–0.80 as substantial, and 0.81–1.00 as almost perfect agreement [36]. The association between prevalence of IBV and NDV in tracheal and choanal cleft swabs from individual birds with IBV and NDV GC in dust was determined by linear regression analysis.

## 3. Results

### 3.1 The profile of IBV and NDV GC in tracheal and choanal cleft swabs and dust samples was affected by days post vaccination

IBV and NDV GC detection rates and viral load at different days post vaccination in tracheal and choanal cleft swabs, and dust samples are summarised in Tables 1 and 2. There was a significant effect of dpv on the IBV GC detection rates and viral load in tracheal swabs, with higher detection rates at 10 and 31 dpv (P < 0.0001). In choanal cleft swabs, IBV detection rates and viral load were highest at 17 and 31 dpv (P < 0.0001). For dust, all 62 collected samples were positive, and log_10_ IBV GC was higher at 31 dpv (Table 1). IBV GC detection rate and viral load were similar in all flocks, except for flock 2 that had a lower IBV detection rate in tracheal swabs (Table 1).

**Table 1:** Proportion of IBV positive samples and log_10_ GC/mg of dust or per swab (LSM ± SEM) in tracheal and choanal cleft swabs and dust samples collected from different flocks at different days post vaccination.

Factors	Tracheal swabs			Choanal cleft swabs			Dust samples		
	N	N. positive/total (%)	Log_10_ IBV GC/swab (LSM ± SE)	N	N. positive/total (%)	Log_10_ IBV GC/swab (LSM ± SE)	N	N. positive/total (%)	Log_10_ IBV GC/mg dust (LSM ± SE)
**DPV**	261	**P < 0.0001**	**P < 0.0001**	480	**P < 0.0001**	**P < 0.0001**	62		**P < 0.0001**
10	21	20/21 (95)^A^	6.90 ± 0.62^A^	-	-	-	14	14/14 (100)	7.89 ± 0.08^B^
17	80	49/80 (61)^B^	4.20 ± 0.32^B^	160	111/160 (69)^AB^	4.75 ± 0.24^A^	16	16/16 (100)	7.64 ± 0.06^B^
24	80	52/80 (65)^B^	4.09 ± 0.32^B^	160	89/160 (56)^B^	3.56 ± 0.24^B^	16	16/16 (100)	7.63 ± 0.06^B^
31	80	72/80 (90)^A^	6.03 ± 0.32^A^	160	132/169 (83)^A^	5.35 ± 0.24^A^	16	16/16 (100)	8.31 ± 0.06^A^
**Flock**	261	**P = 0.02**	***P = 0*.*05***	480	**P = 0.14**	***P = 0*.*06***	62		**P = 0.89**
1	65	52/65 (80)^A^	5.88 ± 0.42^A^	120	82/120 (68)^A^	4.45 ± 0.28^A^	14	14/14 (100)	7.86 ± 0.08^A^
2	65	39/65 (60)^B^	4.40 ± 0.42^A^	120	74/120 (62)^A^	3.98 ± 0.28^A^	16	16/16 (100)	7.84 ± 0.06^A^
3	65	48/65 (74)^AB^	5.16 ± 0.42^A^	120	90/120 (75)^A^	4.84 ± 0.28^A^	16	16/16 (100)	7.86 ± 0.06^A^
4	66	54/66 (82)^A^	5.78 ± 0.42^A^	120	86/120 (72)^A^	4.95 ± 0.28^A^	16	16/16 (100)	7.91 ± 0.06^A^
**Plate location**	**-**	**-**	**-**	**-**	**-**	**-**	62		P = 0.14
1	-	-	-	-	-	-	15	15/15 (100)	7.75 ± 0.07^A^
2	-	-	-	-	-	-	16	16/16 (100)	7.94 ± 0.06^A^
3	-	-	-	-	-	-	15	15/15 (100)	7.94 ± 0.07^A^
4	-	-	-	-	-	-	16	16/16 (100)	7.84 ± 0.06^A^

Bold text indicates statistically significant values (P<0.05), bold text in italics indicate a trend towards significance.

**Table 2:** Proportion of NDV positive samples and log_10_ GC/mg of dust or per swab (LSM ± SEM) in tracheal and choanal cleft swabs and dust samples collected from different flocks at different days post vaccination.

Factors	Tracheal swabs			Choanal cleft swabs			Dust samples		
	N	N. positive/total (%)	Log_10_ NDV GC/swab (LSM ± SE)	N	N. positive/total (%)	Log_10_ NDV GC/swab (LSM ± SE)	N	N. positive/total (%)	Log_10_ NDV GC/mg dust (LSM ± SE)
**DPV**	261	**P < 0.0001**	**P < 0.0001**	480	**P < 0.0001**	**P < 0.0001**	62		**P < 0.0001**
10	21	11/21 (52)^B^	2.58 ± 0.41^C^	-	-	-	14	14/14 (100)	5.89 ± 0.10^C^
17	80	58/80 (73)^B^	3.91 ± 0.21^B^	160	96/160 (60)^B^	3.12 ± 0.17^C^	16	16/16 (100)	6.16 ± 0.09^C^
24	80	74/80 (93)^AB^	5.48 ± 0.21^A^	160	136/160 (85)^AB^	4.82 ± 0.17^B^	16	16/16 (100)	7.46 ± 0.09^B^
31	80	77/80 (96)^A^	5.88 ± 0.21^A^	160	147/160 (92)^A^	5.44 ± 0.17^A^	16	16/16 (100)	8.06 ± 0.09^A^
**Flock**	261	**P = 0.01**	***P = 0*.*06***	**480**	**P = 0.001**	**P < 0.0001**	62		**P = 0.002**
1	65	61/65 (94)^A^	4.99 ± 0.28^A^	120	105/120 (88)^A^	4.99 ± 0.19^A^	14	14/14 (100)	6.98 ± 0.10^A^
2	65	58/65 (89)^A^	4.65 ± 0.28^A^	120	101/120 (84)^A^	4.74 ± 0.19^A^	16	16/16 (100)	7.09 ± 0.09^A^
3	65	49/65 (75)^B^	4.02 ± 0.28^A^	120	82/120 (68)^B^	3.80 ± 0.19^B^	16	16/16 (100)	6.58 ± 0.09^B^
4	66	52/66 (79)^B^	4.20 ± 0.27^A^	120	91/120 (76)^B^	4.31 ± 0.19^AB^	16	16/16 (100)	6.91 ± 0.09^AB^
**Plate location**	**-**	**-**	**-**	**-**	**-**	**-**	62		P = 0.50
1	-	-	-	-	-	-	15	15/15 (100)	6.08 ± 0.09^A^
2	-	-	-	-	-	-	16	16/16 (100)	6.98 ± 0.09^A^
3	-	-	-	-	-	-	15	15/15 (100)	6.93 ± 0.09^A^
4	-	-	-	-	-	-	16	16/16 (100)	6.85 ± 0.09^A^

Bold text indicates statistically significant values (P<0.05), bold text in italics indicate a trend for significance.

NDV GC detection rates and viral load were highest at 31 dpv for choanal cleft and tracheal swabs (P < 0.0001) (Table 2). For dust samples, although there was no difference in the number of NDV GC positive samples among the sampling times, the log_10_ NDV GC was highest at 31 dpv (P <0.0001) (Table 2). NDV detection rates among flocks were different in tracheal (P = 0.01) and choanal cleft swabs (P = 0.001). Log_10_ NDV GC detection in choanal cleft swabs (P < 0.0001) and dust samples (P = 0.002) were the lowest in flock 3, with a tendency towards lower detection in tracheal swabs in this flock (P = 0.05) (Table 2).

### 3.2 IBV and NDV detection rate and viral load in paired choanal cleft and tracheal swabs were similar

Overall, tracheal and choanal cleft swabs had similar IBV GC detection rates with moderate agreement between sample types (McNemar’s = 0.27, Kappa = 0.44). The overall agreement between paired tracheal and choanal cleft swabs in IBV detection rate was 78% (188/240). For discrepant results on paired swabs collected from the same bird, 30 choanal cleft swabs were positive but tracheal swabs negative and 22 tracheal swabs were positive but choanal cleft swabs were negative (Table 3).

**Table 3:** Number of birds positive and negative for IBV and NDV GC in choanal cleft and tracheal swabs.

		IBV			NDV		
		Choanal cleft swabs			Choanal cleft swabs		
		Negative	Positive	Total	Negative	Positive	Total
Tracheal swabs	Negative	37	30	67	15	16	31
	Positive	22	151	173	27	182	209
	Total	59	181	240	42	198	240

Overall, tracheal and choanal cleft swabs had similar NDV GC detection rates with fair agreement between sample types (McNemar’s = 0.09, Kappa = 0.31). There was 82% (197/240) agreement between paired tracheal and choanal cleft swabs in NDV detection rates. For discrepant results on paired swabs collected from the same bird, 27 tracheal swabs were positive but choanal cleft swabs negative and 16 birds were positive in choanal cleft swabs but tracheal swabs negative (Table 3).

Paired tracheal and choanal cleft swabs, collected from same chicken, had excellent agreement in both log_10_ IBV GC (ICC = 1; 4.78 ± 0.19 and 4.94 ± 0.19, respectively) and log_10_ NDV GC (ICC = 0.96; 5.09 ± 0.12 and 4.67 ± 0.12) per swab.

### 3.3 The prevalence of NDV positive birds and log_10_ NDV GC in swabs were positively associated with log_10_ NDV GC in dust but no significant association was found for IBV

**Fig 1:**
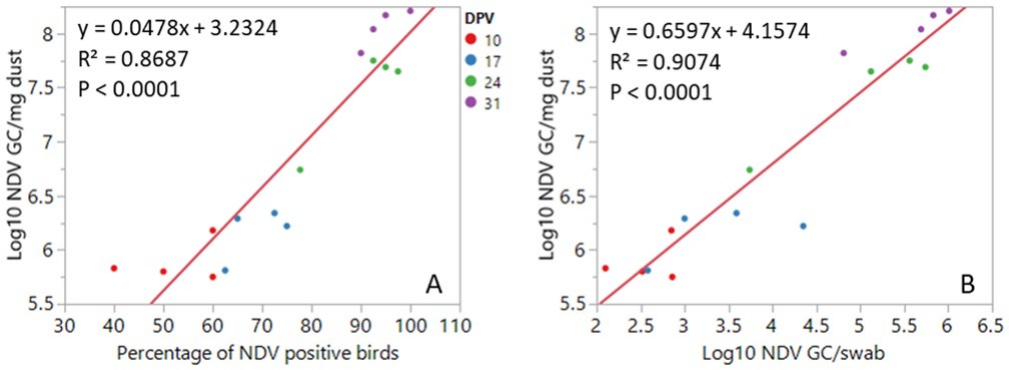
Linear association of log_10_ NDV GC in dust with the percentage of NDV positive birds in swabs (A) and log_10_ NDV GC in swabs (B). Each data point represents the mean value for each one of the four poulty houses on a given dpv.

A bird was considered positive for IBV when either choanal cleft or tracheal swab was IBV GC positive, and the same classification rule applied for NDV. There was a strong positive association between log_10_ NDV GC/mg dust and the prevalence of NDV positive birds and log_10_ NDV GC/swab (Fig 1).

**Fig 2:**
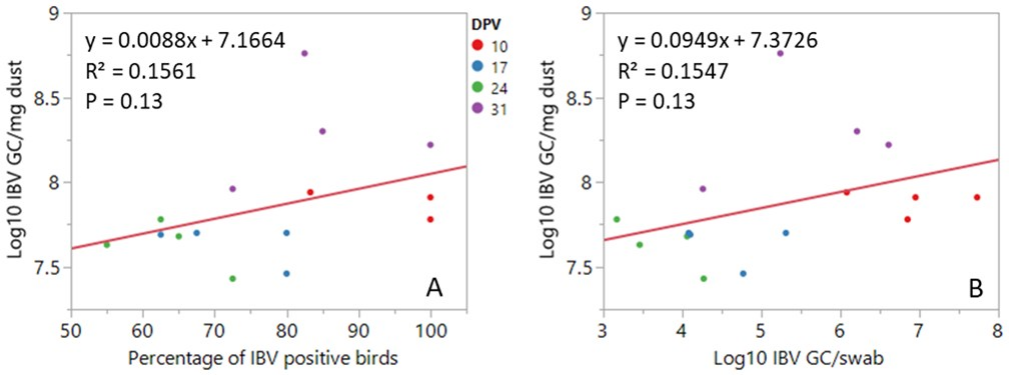
Linear association of log_10_ IBV GC in dust with the percentage of IBV positive birds in swabs (A) and log_10_ IBV GC in swabs (B). Each data point represents the mean value for each one of the four poulty houses on a given dpv.

The association of log_10_ IBV GC/mg dust with both percentage of IBV positive birds and log_10_ IBV GC/swab was not significant (Fig 2). This poor association is probably due to the high loads of log_10_ IBV GC in dust despite a significant reduction in the log_10_ IBV GC load and detection rates in swabs from 10 to 24 dpv (Table 1).

### 3.4 Location of dust collection affected dust deposition rate but not IBV and NDV GC load

The amount of dust collected was affected by both dpv (p = 0.0001) and location of settle plates in the house (p < 0.0001). Dust deposition rate was highest near to the extraction fan end of the poultry house (10.37 ± 0.94 mg/100 cm^2^/day) compared to plate farthest from the extraction fan (6.67 ± 0.97 mg/100 cm^2^/day). Dust deposition rate increased with bird age, which was 2.8 ± 0.47 mg/100 cm^2^/day for days 0 to 9 and 10.92 ± 0.44 mg/100 cm^2^/day for 10–16 days of bird age. Then after it remained higher until the last collection at day 31. Although there was a difference in dust deposition rate at different locations in the house, there was no difference in viral load for both IBV (p = 0.30) and NDV (p = 0.62).

## 4. Discussion

In this study, the IBV and NDV GC profiles in dust and choanal cleft and tracheal swab samples after vaccination with live vaccines were determined in commercial meat chicken flocks. There was a fair to moderate agreement on viral detection between tracheal and choanal cleft swabs for both viruses, with no bias for a higher detection in a particular sample type. IBV and NDV GC were readily detected in dust samples at 10 dpv until the end of study at 31 dpv. The findings from this study indicate that dust could be used as a screening tool for IBV and NDV in commercial flocks.

A high IBV GC load was detected in dust samples and tracheal swabs at 10 dpv with a subsequent decline in the detection rates and GC load in individual birds at 17 and 24 dpv, followed by increase in GC load at 31 dpv. This profile is in agreement with previous studies that reported vaccine virus shedding from 3 dpv, and a peak in viral load and detection rates at 11 and 14 dpv [13, 14, 37]. Later increase in the viral load could be due to re-excretion of the virus from persisting infection. In contrast to IBV, NDV GC detection in swabs and dust increased with bird age. This may be explained by the longer incubation period of NDV for mild strains [9]; or by a rolling infection due to poor initial vaccine uptake that could have occurred because of the presence of maternal antibodies that prevented initial infection or because of sub-optimal vaccine administration. As serological tests were not performed to evaluate the presence of maternal antibodies at hatch and seroconversion after vaccination, it was not possible to further investigate the reasons for the increase in NDV detection rates over time.

IBV and NDV GC were readily detected in dust samples after vaccination. The detection of virus GC in dust in early sampling days could be partially explained by aerosalisation of the vaccine virus deposited in the feathers of the birds during spray vaccination. However, the sustained high levels of IBV and NDV GC in dust and the high proportion of positive birds throughout the cycle suggest that at least part of the detected viral load was due to active IBV and NDV shedding. In a previous study, a high infectious laryngotracheitis virus GC load was reported in dust at 7–8 days post drinking water vaccination when the percentage of positive birds in laryngotracheal swabs was as low as 25% [28, 38]. Another study reported that infectious laryngotracheitis virus GC load in dust remained similar while the percentage of positive birds in tracheal swabs declined from 100% to 58% [29]. This is likely explained by accumulation of viral genome in poultry dust over time. Further work is required to determine the contributions of dried vaccine virus in feathers after coarse spray vaccination and active virus shedding by infected birds in the IBV and NDV GC load detected in dust.

Under the conditions of this study, there was a strong positive association of individual bird measures (prevalence of positive birds and viral load in swabs) for NDV and log_10_ NDV GC in dust. This indicates that monitoring of NDV using dust samples may be a good proxy for NDV shedding in individual chickens throughout the production cycle. Unlike NDV, there was a weak positive association between IBV GC detection rates and viral load in swab samples and in dust. The differences on the NDV and IBV profile are likely because of the high proportion of NDV positive birds throughout the study, while the decrease of IBV positive birds was not accompanied by a decrease in viral load in dust samples as discussed above. A total of 741 swabs and 62 dust samples were processed in this study. A single dust sample at each sampling point could identify flocks as positive for IBV or NDV when at least 40% of the chickens were positive while a larger number of swab samples were required to account for lower detection rates. Collecting swab samples require handling of individual birds which is labour intensive and stressful for both birds and staff. Poultry dust, a population level sample, is easy to collect, welfare friendly and can be used as a screening tool for IBV and NDV GC in meat chickens. This study provides proof of concept that dust can be used as a population level for detecting IBV and NDV GC in poultry houses and this approach could potentially be used to monitor vaccine uptake or the incursion of field strains in commercial flocks. Further studies with larger numbers of flocks, with earlier days of sample collection and a follow up with a serological testing of the flock are required for further validation of this work to be used for assessing vaccine uptake.

Choanal cleft and tracheal swabs were comparable in detecting IBV and NDV GC following live vaccination. Therefore, easier to collect choanal cleft swabs could be used for sampling of individual birds without loss in sensitivity. Collection of tracheal swabs requires a higher skill level and has a higher risk of bird stress compared to collection of choanal cleft swabs. This finding brings practical information to veterinarian practioners and researchers evaluating IBV and NDV shedding profiles with potential increased bird welfare if choanal swabs are selected.

Dust deposition rate was affected by location in the house and bird age. All houses in this study were tunnel ventilated and the highest amount of dust was collected in plates located near exhaust fans which is in agreement with previous studies [28, 29]. An increase in dust deposition rate with bird age in this study is consistent with a previous report in layer chickens [29]; but different from another study which reported a decline in dust deposition rate with bird age [28]. This disparity between studies may be because of differences in the bedding material and level of ventilation used. IBV and NDV GC were similar for dust samples collected from different locations within the poultry house in agreement with previous reports for infectious laryngotracheitis virus and Marek’s disease virus [28, 29]. Dust samples for detection and quantification of IBV and NDV GC can be collected from any location within a poultry house.

## 5. Conclusions

In conclusion, detection rate and load of IBV and NDV genomes following spray vaccination at hatchery in meat chickens grown in tunnel ventilated houses can be determined using tracheal and choanal cleft swabs, and dust samples. Choanal cleft swabs, which are easier and less invasive to collect compared to tracheal swabs, gave comparable results to tracheal swabs for detection of IBV and NDV GC and are thus a preferable sample type. Dust could be used for monitoring IBV and NDV GC in poultry houses. NDV GC in dust samples were positively associated with the detection rates and viral load in swabs from individual birds for the duration of the production cycle. Higher level of IBV in dust 10 dpv was likely due to earlier shedding of this virus and the possibility of detecting the virus earlier than 10 dpv. Dust samples for detection and quantification of IBV and NDV GC can be collected from any location within poultry house, with increased dust deposition near to exhaust fans in tunnel ventilated houses.

## Supporting information

S1 Raw data (XLSX)
